# Unraveling the diversity and functional potential of cyanosphere microbiomes assembled from terrestrial cyanobacteria

**DOI:** 10.1128/aem.01043-26

**Published:** 2026-07-20

**Authors:** Brianne Palmer, Estelle M. Couradeau, Jeffrey R. Johansen, Tania Kurbessoian, Jose Ortega Carranza, Jason E. Stajich, Ryan Ward, Nicole Pietrasiak

**Affiliations:** 1 Bonn Institute of Organismic Biology, University of Bonn 9374, Bonn, Germany; 2 Department of Ecosystem Science and Management, The Pennsylvania State University 8082, University Park, Pennsylvania, USA; 3 The Huck Institutes of the Life Sciences, The Pennsylvania State University 8082, University Park, Pennsylvania, USA; 4 Biology Department, John Carroll University 2566, University Heights, Ohio, USA; 5 Faculty of Sciences, Botany Department, University of South Bohemia, České Budějovice, Czech Republic; 6 Department of Microbiology and Plant Pathology, University of California-Riverside, Riverside, California, USA; 7 Plant and Environmental Sciences Department, New Mexico State University 4423, Las Cruces, New Mexico, USA; 8 School of Life Sciences, University of Nevada — Las Vegas 14722, Las Vegas, Nevada, USA; Georgia Institute of Technology, Atlanta, Georgia, USA

**Keywords:** biological soil crust, wet walls, soil, deserts, tropics, subaerial

## Abstract

The cyanosphere is composed of non-cyanobacterial microorganisms living within the exopolysaccharide sheath of cyanobacteria, interacting with the cyanobacterial hosts and their surrounding environment. Understanding the interactions between cyanobacteria and their cyanospheres can help predict the success of terrestrial cyanobacteria in providing ecosystem services in nutrient-poor environments. However, knowledge of the microbial diversity and functions within the cyanosphere remains limited. Here, we used metagenomic sequencing to reconstruct 415 metagenome-assembled genomes (MAGs) from cyanosphere-associated microbes linked to 56 terrestrial cyanobacteria cultures, representing 12 cyanobacterial orders. Our findings showed that the composition of cyanosphere microbial communities was significantly shaped by environmental factors such as habitat of host origin, including precipitation and temperature. Three microbial genera, *Brevundimonas*, *Devosia*, and *Sphingopyxis*, were present in over 30% of the cyanospheres, suggesting a core cyanosphere microbiome. Functional gene analysis showed a distinction between the cyanobacteria and their associated cyanospheres, with dissimilatory nitrate reduction being the dominant pathway in the cyanosphere, an anaerobic process that retains nitrogen in the host-cyanosphere system in contrast to denitrification. While nitrogen fixation was more common in the cyanobacteria, 15 cyanospheres also contained nitrogen fixation genes, including in hosts that were nitrogen fixation capable themselves. The cyanosphere also contained genes for polysaccharide lyases, indicating a possible link to the exopolysaccharides produced by the cyanobacteria. Given the observed variability in microbial community composition and function across different cyanobacterial hosts, future ecological assessments and restoration efforts involving cyanobacteria should not only focus on the cyanobacteria themselves but also consider their associated microbial communities.

**IMPORTANCE:**

Our study identifies members of an understudied and under-valued microbial community, the cyanosphere. We used a diversity of terrestrial cyanobacteria to understand how the cyanosphere composition and predicted functions were influenced by the host cyanobacterium and environmental factors using metagenomics. This is a novel approach to studying the cyanosphere, providing insights into the diversity of terrestrial microbial communities. Importantly, our results underscore the need to consider microbial consortia when assessing the ecological potential of cyanobacteria in terrestrial restoration.

## INTRODUCTION

Cyanobacteria are prevalent and perform various ecosystem functions in almost any environment where sunlight is at least temporarily available. This includes terrestrial environments, which are highly complex and are characterized by often very contrasting abiotic and biotic conditions, such as found in soil, hydroterrestrial, and subaerial habitats (1, 2). In soils, cyanobacteria contribute to carbon sequestration (3) and enrich soil fertility by fixing atmospheric nitrogen and reducing soil erosion (4, 5). Cyanobacteria are also foundational components of biological soil crusts, which cover approximately 30% of drylands globally (6). In hydroterrestrial environments, such as periodically dry vernal pools, algal and cyanobacterial biofilms often form rapidly after rehydration and are important for maintaining local food webs as microscopic primary producers (7, 8). In subaerial habitats, which include exposed surfaces like rocks and tree bark, cyanobacteria are pioneers, contributing to the formation of epilithic and epiphytic biofilms that support other microbial and plant life (9–11). As early colonizers on these substrates, the cyanobacteria can enrich the substrate with organic carbon through photosynthesis and can improve nutrient cycling in the micro-environment by trapping dust and soil particles and fixing atmospheric nitrogen (10, 12).

A distinctive feature of many cyanobacteria is the presence of the cyanosphere (13). The cyanosphere is a microenvironment surrounding cyanobacterial cells, especially in filamentous forms, and is inhabited by microorganisms that closely associate with their cyanobacterial hosts (13). These microorganisms may benefit from the organic compounds produced by the cyanobacteria through photosynthesis, and in return, they can assist the cyanobacteria by providing essential nutrients (14). Previous investigations of the cyanosphere revealed a diverse set of organisms inhabiting non-axenic cyanobacterial cultures. Isolated cyanobacterial strains typically coexist with heterotrophic bacterial partners, indicating that these associations are common and perhaps essential to cyanobacterial culture stability (15).

Cyanosphere studies typically rely on investigations of cultured cyanobacteria, and many isolates are sequenced soon after initial purification to minimize the development of a laboratory-adapted “culturosphere” (16). However, there is evidence that the culturosphere largely originates from, and retains features of, the naturally occurring cyanosphere, which hints to a microbiome that may depend on or support the health of the host cyanobacteria. Although axenic cyanobacterial cultures can be maintained, they are generally less stable and robust than those containing coexisting bacterial populations (17). Several studies have successfully used xenic culture collection strains to characterize cyanobacterial-associated microbiomes (18–20), and frequent transfers combined with gentle mechanical cleaning can maintain communities dominated by organisms tightly associated with cyanobacteria (21). Recent work further supports this interpretation. Kust et al. demonstrated that model cyanobacterial strains repeatedly recruit highly similar sets of noncyanobacterial taxa when cocultured with natural freshwater microbial inocula, forming stable laboratory consortia with conserved taxonomic and functional features (22). These findings indicate that the microbiota detected in non-axenic cyanobacterial cultures often reflect ecologically meaningful associations derived from the cyanosphere rather than incidental laboratory contamination.

Using metagenomic and metaproteomic approaches, it has been demonstrated that interactions between photoautotrophs and heterotrophs are largely governed by mutualistic relationships (23, 24). Environmental factors such as nutrient availability, light, and moisture can alter metabolic exchange and interaction strength between photoautotrophs and heterotrophs, underscoring the role of ecological context in shaping cyanosphere structure (21, 22). These studies highlight the ecological complexity, functional diversity, and interdependence that characterize the cyanosphere and its associated microbial communities.

Current research on the terrestrial cyanosphere is limited. Previous work on the cyanosphere has focused on *Microcoleus vaginatus*, a filamentous cyanobacterium known to be an early colonizer of biological soil crusts without the ability to fix nitrogen. In one of the first cyanosphere studies, Couradeau and her colleagues found that most of the inhabitants of the *M. vaginatus* cyanosphere were copiotrophs, and some were diazotrophs and contained more nitrogen-fixation genes in comparison to the bulk soil microbiome (13). This highlights the potential role of nutrient exchange of carbon and nitrogen between the cyanobacteria and the diazotrophs in the cyanosphere. A subsequent study found evidence of a carbon-nitrogen exchange between *M. vaginatus* and the cyanosphere (14). The success of *M. vaginatus* as an early colonizer and important species for cyanobacterial-mediated dryland soil restoration may be because of the bacteria within the cyanosphere, such as *Arthrobacter, Massillia,* and *Bacillus* spp., which, when added to the cyanobacterial growth medium, increased cyanobacterial growth (25). Nelson and his colleagues found evidence of signaling between *M. vaginatus* and its cyanosphere, further emphasizing the interconnectedness between the cyanobacteria and their associated microorganisms (26)

Beyond *M. vaginatus,* a few studies have assessed the microbial community composition within the cyanosphere, with most studies focused on either soil or freshwater habitats. In a direct comparison of the rhizosphere and the cyanosphere, another study examined growth-promoting strains in both the rhizosphere and biocrust cyanospheres. They found 18 phyla common to both and identified growth-promoting isolates such as *Bosea* and *Pseudoarthrobacter* in the cyanosphere (27). Within aquatic environments, one study used metatranscriptomics to examine the cyanospheres of both nitrogen-fixing and non-nitrogen-fixing aquatic cyanobacteria, *Dolichospermum* and *Microcystis,* showing that the bacterial associations were determined by the cyanobacterial host, with the most abundant phyla being Bacteroidetes, Gemmatimonadetes, and Proteobacteria. They also identified functional redundancy within the cyanosphere (28). However, the diversity and ecology of hydroterrestrial and subaerial habitats are virtually unexplored.

Based on the limited research of terrestrial cyanospheres, we know that the identity of the microbes within the cyanosphere can be important for the success of the cyanobacteria (25), and the functions of the cyanosphere microbes can provide essential nutrients to the cyanobacteria (13, 14, 26). However, the breadth of terrestrial cyanobacterial species is large, and current research is limited to *M. vaginatus*. Therefore, there is a need to understand the identity and function of the cyanosphere microbes within the cyanosphere from a diverse set of terrestrial cyanobacteria.

The use of next-generation sequencing techniques allows for the identification of the microbial community within the cyanosphere. Metagenomics, in particular, provides information about the identity of the microbes and the potential functions they may be performing. Here, we used metagenomic sequencing on 56 cultured terrestrial cyanobacteria and their cyanosphere microbiomes. These cyanobacteria are classified into 12 of the currently recognized 19 orders in cyanobacteria and represent broad phylogenetic diversity. We sought to understand how the taxonomy of the host cyanobacteria and the environment affects the bacterial community of the cyanosphere.

## MATERIALS AND METHODS

### Cyanobacterial cultures

Metagenomes were obtained from 56 unialgal polycultures containing cyanobacteria and associated cyanosphere microbiomes. Polycultures were established via traditional dilution plating of environmental samples obtained from various collection trips led by Johansen, Pietrasiak, and collaborators. Collection years and detailed information can be found in Ward et al. (2) and in Table S1. Polycultures were isolated between 1992 and 2015, with one additional culture from another collection made in 1965, and represent reference cultures of recently well-studied taxonomic and phylogenetic benchmarks in cyanobacteria taxonomy and systematics. The dominant cyanobacterial members in these cultures are phylogenetically diverse, spanning 12 orders of cyanobacteria. Cultures have been maintained in two culture collections at John Carroll University (JCU) and the University of Nevada—Las Vegas (UNLV) on solid or liquid Z8 medium, an oligotrophic freshwater algal medium (29). Although dates of isolation span 50 years, close to identical culture conditions (use of Z8 medium, 16/8 h light/dark photoperiod, low light intensity, grown in climate-controlled chambers) have been maintained at JCU since 1992 and have been reproduced at UNLV. At the time of DNA extraction, the cultures’ microbial biomass was dominated by cyanobacteria.

### Metagenomic sequencing

Polyculture biomass was grown in liquid Z8 medium, harvested, flash-frozen, and stored before DNA extraction using the Qiagen DNeasy PowerLyzer kit with bead-beating, following detailed protocols available on protocols.io (dx.doi.org/10.17504/protocols.io.brg4m3yw) (2). Extracted DNA was stored at −20°C prior to shipment to the Joint Genome Institute (JGI). JGI performed the library preparation and next-generation Illumina sequencing steps as follows. DNA library preparation was performed using the KAPA Biosystems high-throughput library preparation kit p/n KK8235 on a PerkinElmer Sciclone next-generation sequencing (NGS) robotic liquid handling system. Then, 200 ng of sample DNA was sheared to 300 bp using a Covaris LE220 focused ultrasonicator. The sheared DNA fragments were size-selected by double solid-phase reversible immobilization (SPRI). The selected fragments were end-repaired, A-tailed, and ligated with Illumina-compatible sequencing adaptors from IDT containing a unique molecular index barcode for each sample library. The prepared libraries were quantified with a KAPA Biosystems quantitative PCR (qPCR) kit on a Roche LightCycler 480 real-time PCR instrument. Genomic libraries were sequenced with a NovaSeq instrument (Illumina, San Diego, CA) using NovaSeq XP V1 reagent kits and an S4 flow cell following a 2 × 150 bp indexed run recipe. Demultiplexed reads were processed with BBDuk v38.87 (17) to remove contaminants, trim adapter sequence and “G” homopolymers ≥5 in size at the ends, quality trim reads, and remove reads with ≥4 “N” bases, with an average quality score of >3, or with a length of <51 bp. Raw sequence products were submitted to NCBI SRA archive. A list of BioProjects and SRAs is provided in Ward et al. (2) with additional descriptions in Table S1.

### Microbial community analyses

Sequence Read Archive (SRA) reads were first uploaded into the KBase platform (30). The quality of these reads was assessed using the Fast-QC tool (31), and those that did not meet the quality threshold (*Q* score >30) were refined using Trimmomatic (32). The reads for each cyanobacterium were assembled using metaSPAdes (33). The assembled contigs were then segregated into Metagenome-Assembled-Genomes (MAGs) via MaxBin2 (34). All MAGs were dereplicated using DAS Tools (35). MAGs that did not meet the minimum requirements for medium-quality MAGs (>50% completion and <10% contamination) (36) were removed (35 MAGs). Taxonomic classification was conducted using GTDB-tk (37). A taxonomy table, incorporating DRAM annotation from KBase (DRAM-lite), was generated to establish a connection between taxonomy and function (38). DRAM-lite produces four functional modules: (i) carbon utilization, including CAZyme annotations and pathways for polysaccharide and organic carbon degradation; (ii) organic nitrogen metabolism; (iii) energy metabolism, including nitrogen and sulfur cycling, respiration, and electron transport pathways; and (iv) tRNA predictions. These DRAM module outputs were used to assess the metabolic potential of bacteria associated with cyanobacterial cultures, with particular emphasis on carbon degradation and nitrogen cycling pathways. The KBase narrative is publicly available at https://narrative.kbase.us/narrative/202318.

All subsequent analyses were performed in R (version 4.3.1). The *amp_alpha_diversity* function within the R package *ampvis2* was used to calculate alpha diversity metrics, including the total number of MAGs and Shannon diversity (39). Differences in MAG richness and Shannon diversity between categorical variables were calculated using ANOVAs with Tukey HSD post-hoc tests.

To determine if there is a core cyanosphere, we calculated the number of cyanospheres in which a bacterial genus occurred. Genera found in >30% of the cyanospheres were classified as part of the core cyanosphere. The package *microeco* was also used to create a heatmap of the most abundant genera.

Bray-Curtis distances were used to calculate the beta diversity using the *microeco* package. To assess differences in the microbial community composition across categorical variables, a PERMANOVA analysis was performed using the *cal_manova* function. Beta diversity was visualized through Principal Coordinates Analysis (PCoA) plots generated in the *microeco* R package (40).

Climate variables were obtained from BIOCLIM (41). When latitudinal and longitudinal data were available, we identified the mean annual temperature and the annual precipitation at the collection location of each cyanobacterium in BIOCLIM. Due to the covariance between environmental variables, only mean annual temperature and precipitation were used. The impact of continuous variables such as temperature, precipitation, and altitude on microbial community composition was evaluated using the *cal_ordination_envfit* function within *microeco*. This relationship was further illustrated with a distance-based Redundancy Analysis (dbRDA) plot using the *microeco* R package (40). The functional abundances, as assigned via DRAM, were visualized using heat maps created with the *pheatmap* R package (42) for the cyanosphere community and the host cyanobacteria. The code for this project is publicly available on GitHub at https://github.com/briannepalmer/Cyanosphere.

## RESULTS

Including the host cyanobacteria MAGs, 472 MAGs were retained. Of those, 402 qualified as “high quality” MAGs with >90% completion and <5% contamination (Table S2). Metagenomic analysis indicated that each culture (*n* = 56) contained a single cyanobacterial genome. In total, we recovered 415 non-cyanobacterial MAGs associated with the cyanobacteria.

### Geographic representation

**Fig 1:**
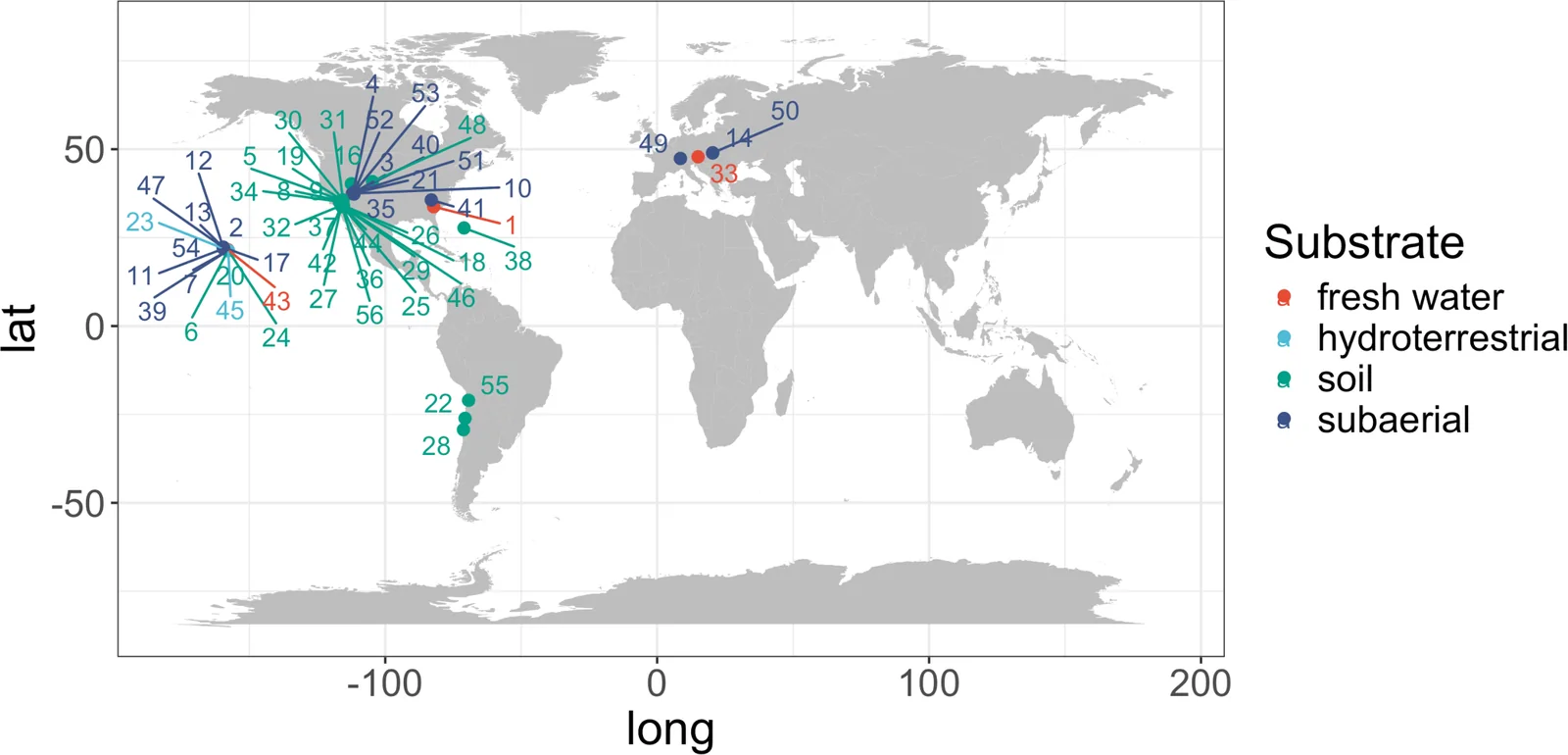
Map of the locations where the cyanobacteria were collected. One cyanobacterium (*Drouetiella lurida*) did not have coordinate data and was not included on the map. The points are colored by the habitat where the cyanobacteria were collected, and the numbers are assigned to each cyanobacterium found in Table S1. The map was created in R using the packages ggplot2 and ggmap.

The 56 cyanobacterial hosts represented 14 locations spanning 3 continents (Fig. 1). Soil cyanobacteria were collected from five types of parent material (dolomite, granite, gypsum, gneiss, and limestone) (Table S1). The altitude of the host cyanobacteria collection location ranged from 11 to 2,043 m. The annual precipitation was between 3 mm and 3,229 mm. The annual mean temperature ranged from 5.2°C to 23.6°C.

#### Core and most abundant genera

**Fig 2:**
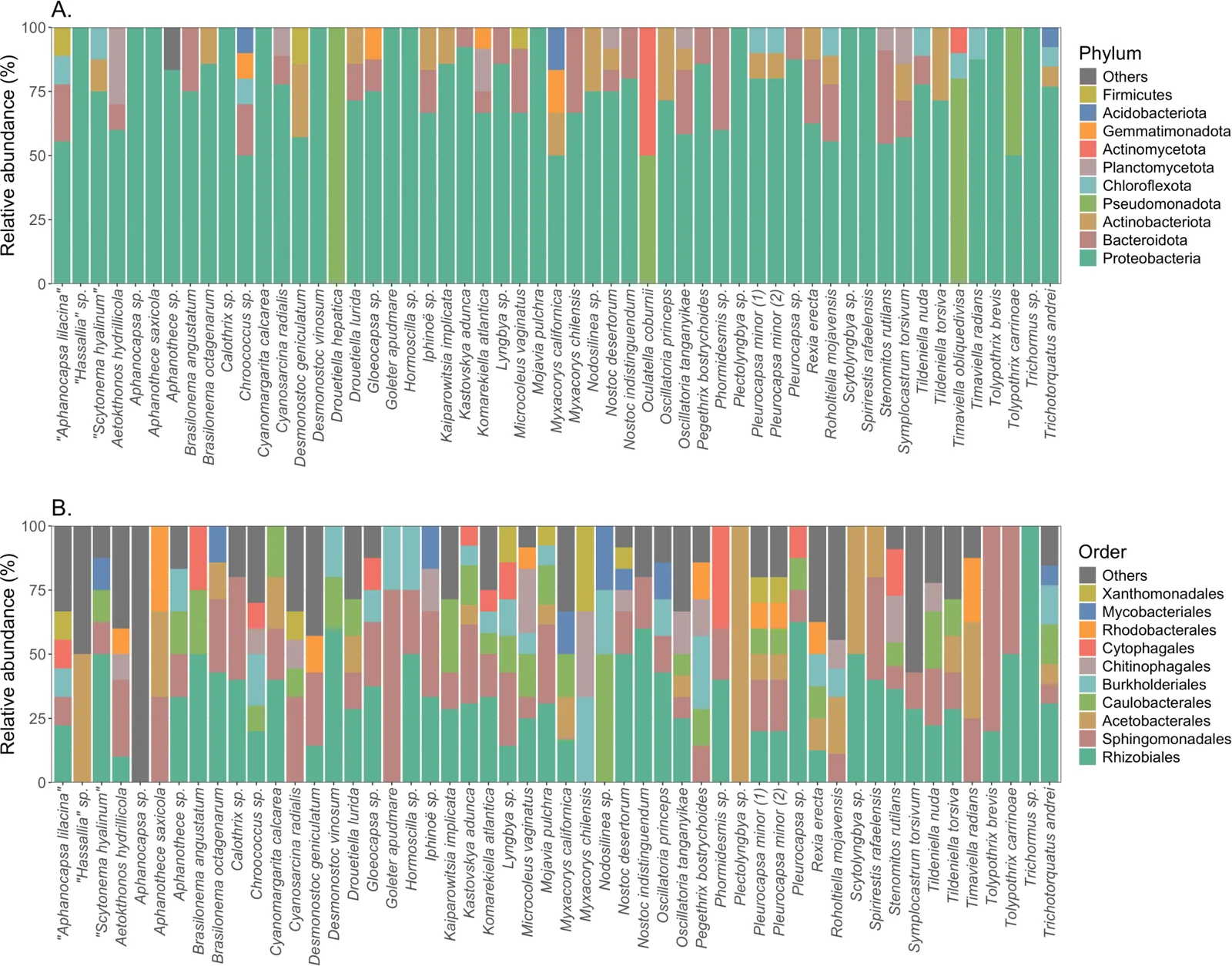
Bar plots depicting the relative abundance of the most abundant phyla (**A**) and the Proteobacteria orders (**B**) for the MAGs in the cyanosphere.

Proteobacteria (73.7%), Bacteroidota (10.6%), and Actinobacteria (5.5%) were the most abundant phyla in the cyanospheres (Fig. 2A). Within Proteobacteria, Rhizobiales was the most abundant order, occurring in 69% of the cyanospheres (Fig. 2B).

**Fig 3:**
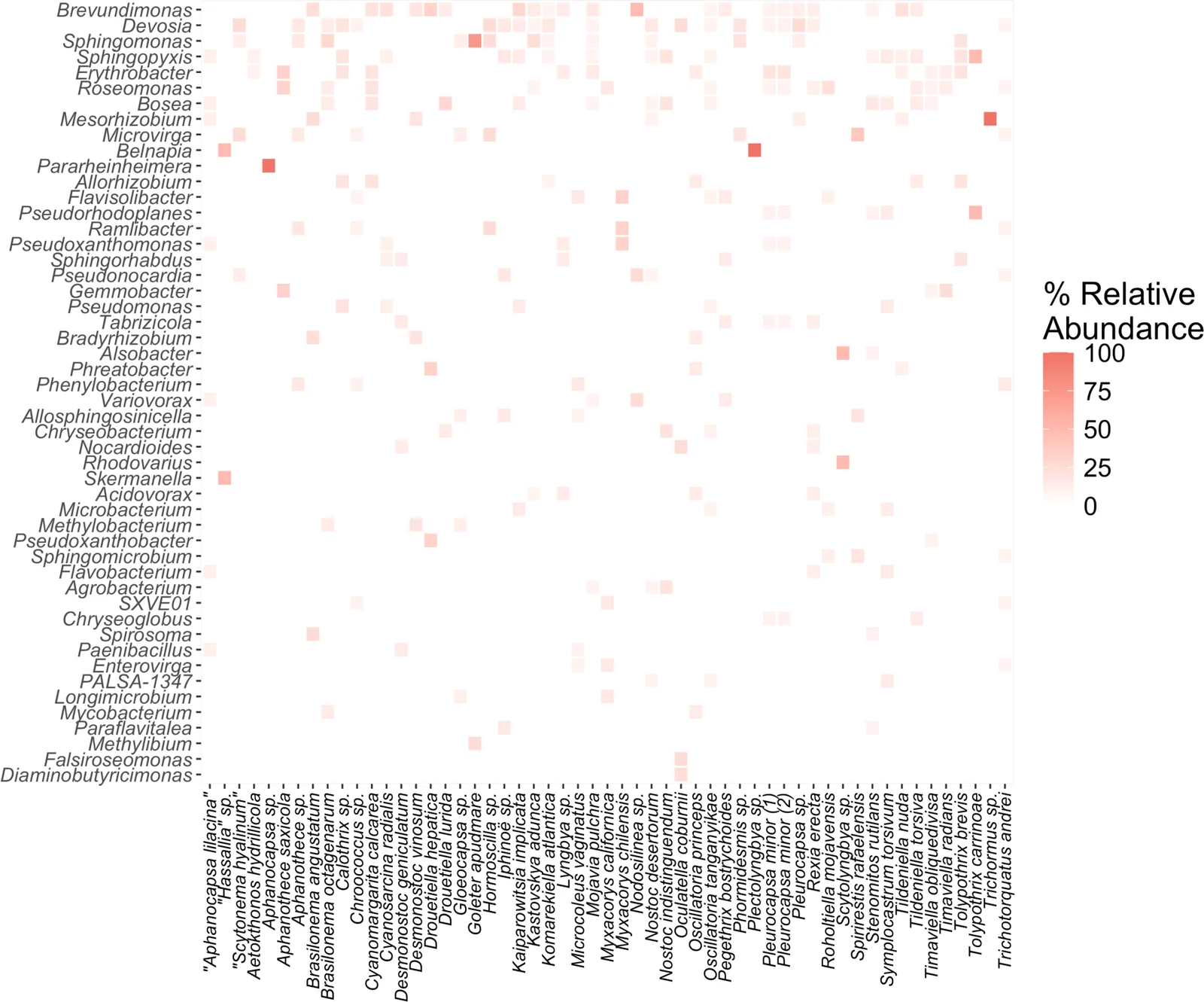
Heatmap showing the relative abundance of the 50 most abundant non-cyanobacterial genera found within each cyanobacterial host. Dark red indicates higher relative abundance, while light red and white indicate low or no abundance in the given cyanosphere. The genera on the *y*-axis are ordered by the total relative abundance across all cyanospheres (*x*-axis).

Only three genera were found in >30% of the samples: *Brevundimonas* (37.5%), *Devosia* (35.7%), and *Sphingopyxis* (30.5%)(Fig. 3). These genera could represent a core terrestrial cyanobacterial cyanosphere. The most abundant genera were *Brevundimonas*, *Sphingomonas*, and *Devosia*, which were also found in cultures originating from three terrestrial habitats (soil, subaerial, hydro-terrestrial). Two genera, *Allorhizobium* and *Sphingopyxis*, were found in cyanospheres from all four habitat types.

#### Alpha diversity metrics were consistent across cyanobacterial hosts

**Fig 4:**
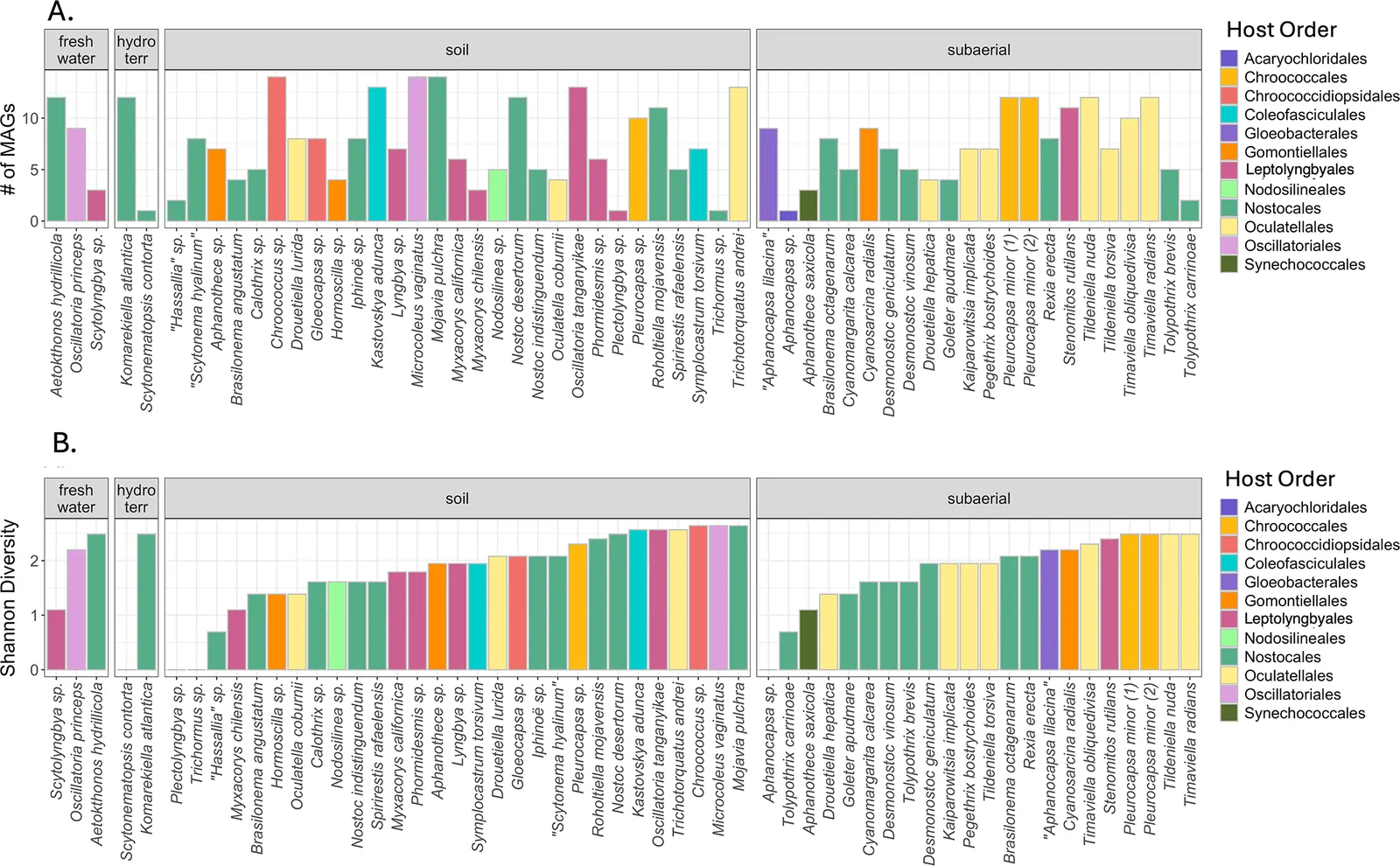
Bar charts depicting the total number of MAGs (**A**) and the Shannon diversity of the MAGs (**B**) for each cyanosphere, grouped by habitat. The bars are colored by the order of the host cyanobacteria. “hydroter” denotes “hydroterrestrial.”

MAG richness and Shannon diversity did not vary among cyanobacterial orders (ANOVA, *P* = 0.18, *P* = 0.09) or among host-origin habitats (ANOVA, *P* = 097, *P* = 0.71) (Fig. 4). *Chroococcus* sp., *Microcoleus vaginatus*, and *Mojavia pulchra*, each with 14 identified cyanosphere members, contained the most MAGs. *Aphanocapsa* sp., *Plectolyngbya* sp., *Scytonematopsis contorta*, and *Trichormus* sp. only contained one non-cyanobacterial MAG. There was an average of 7.4 MAGs per cyanobacteria. The cyanobacteria host *Pelatocladus maniniholoensis* did not have any identifiable MAGS in the cyanosphere.

#### Microbial community composition varies between habitats, rather than between cyanobacterial hosts

**Fig 5:**
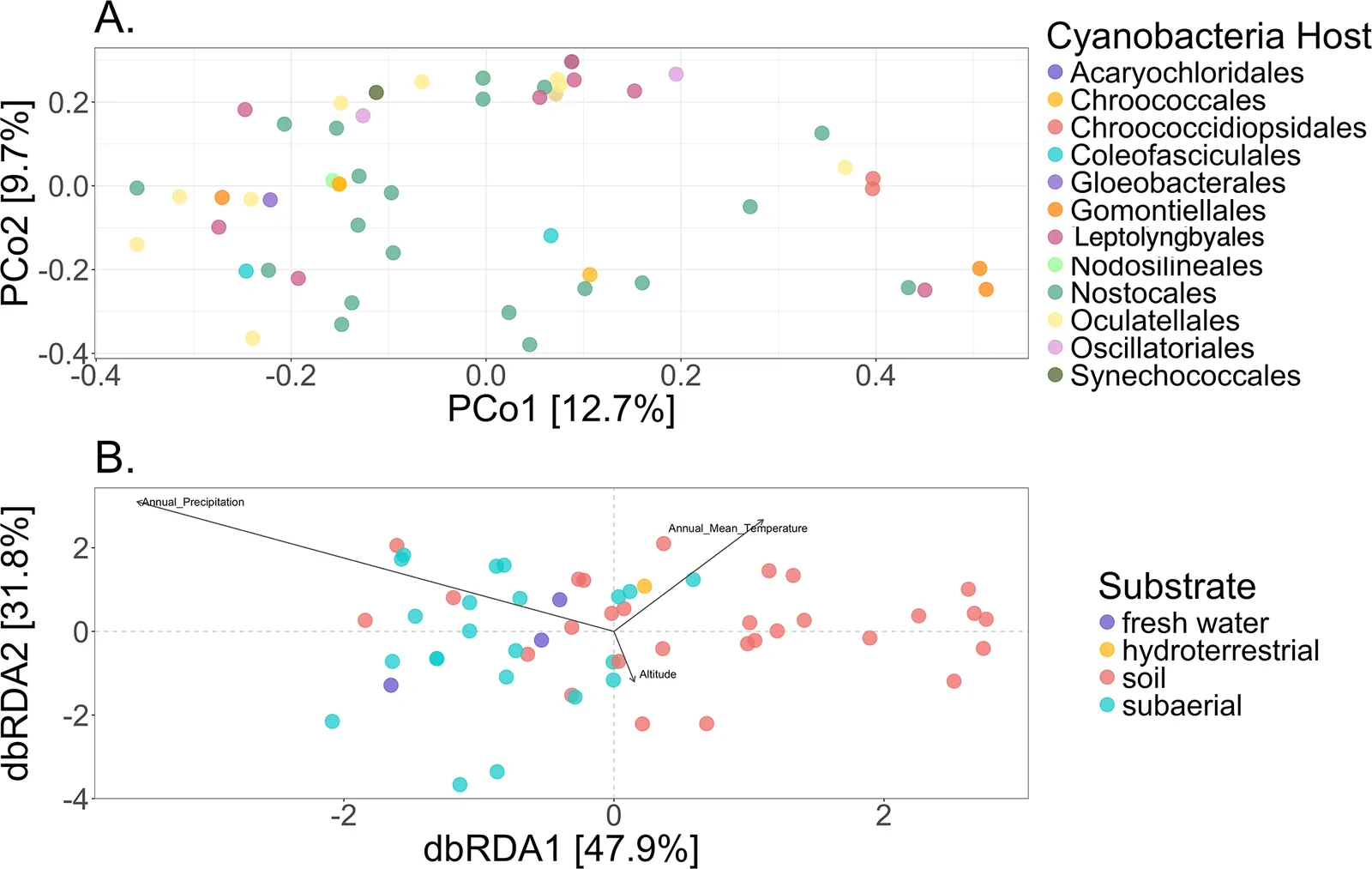
Ordination plots showing the community composition based on the MAG genera in the cyanospheres. (**A**) A PCoA depicting the MAG community composition colored by the order of the cyanobacterial host. (**B**) An RDA plot colored by habitat, indicating the grouping of the cyanosphere community composition. The ellipses show groupings of the cyanospheres with more than three representatives. The arrow vectors (Annual Precipitation, Annual Mean Temperature, and Altitude) show environmental variables that were significantly (*P* < 0.05) correlated with the cyanosphere community composition.

Based on the PCoA ordination modeling and PERMANOVA (*P* = 0.19), there were no differences in the bacterial community composition among cyanobacterial host orders. The first two PCoA axes explained 12.7% and 9.7% of the variation in the community composition, and there was little clustering between bacterial communities with the same cyanobacterial host order (Fig. 5A). However, the microbial community composition did vary by host genus (PERMANOVA, *P* = 0.035). There was also a significant effect of the habitat of host origin on the community composition (PERMANOVA, *P* = 0.004). The mean annual temperature (*P* < 0.001), annual precipitation (*P* < 0.001), and altitude (*P* = 0.006) where the cyanobacteria were collected also influenced the community composition (Fig. 5B). When the environmental factors and habitat type were included in an RDA ordination model, there was a distinct grouping between the cultures originating from the soil and from subaerial sites, and the axes explained 45.3% and 36% of the variation in the microbial community composition. The cultures originating from soil were grouped with the annual mean temperature vector, while the subaerial samples were grouped with annual precipitation (Fig. 5B).

To complement the MAG-based analysis, we performed taxonomic classification of unassembled reads using Kaiju. This approach provides an assembly-independent overview of community composition. The Kaiju results confirmed that *Brevundimonas* and *Sphingopyxis* were the most abundant taxa across samples, consistent with the dominant genera recovered in the MAG data set (Fig. S1). However, unlike the MAG-based community profiles, Kaiju-derived taxonomies did not show significant effects of habitat of host origin or host phylogeny (PERMANOVA, *P* = 0.15 [habitat], *P* = 0.22 [host order], Fig. S2). This likely reflects the lower taxonomic and ecological resolution of short-read classification compared to MAG reconstruction. Therefore, Kaiju results are included as a validation of overall diversity patterns but were not used for functional or ecological interpretation.

#### Cyanosphere and cyanobacterial hosts differ in key functions

**Fig 6:**
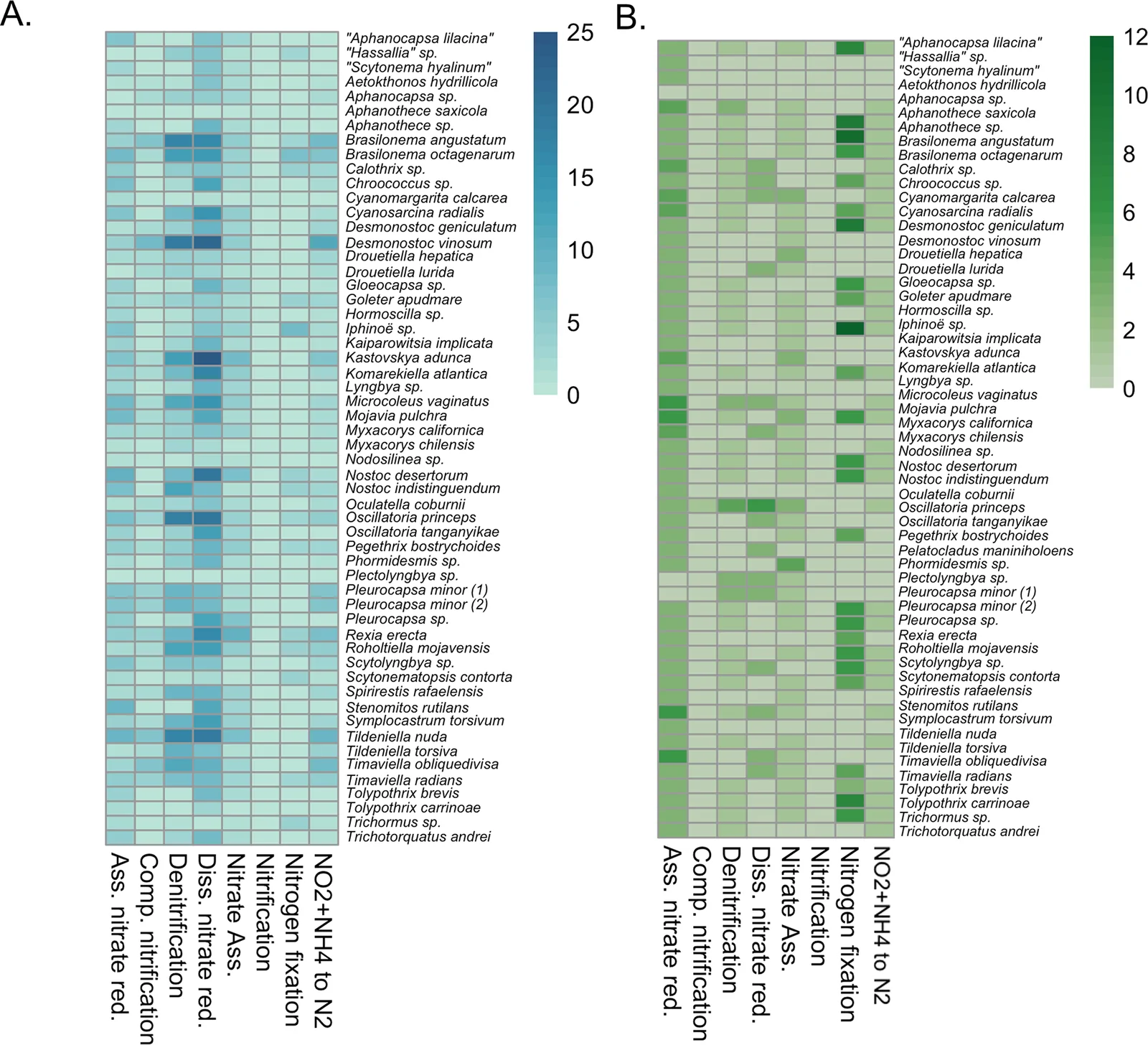
Heatmaps depicting the abundance of genes relating to nitrogen pathways for the cyanosphere (**A**) and cyanobacteria (**B**). The *x*-axis labels describe the nitrogen pathways, including assimilatory nitrate reduction, complete nitrification, denitrification, dissimilatory nitrate reduction, nitrate assimilation, nitrification, nitrogen fixation, and nitrite + ammonia => dinitrogen. Pathway presence was defined according to DRAM’s marker-based criteria.

Pathway presence within the cyanosphere microbes and cyanobacteria hosts was assessed using DRAM’s marker-based criteria, in which a metabolic pathway is considered present if all required diagnostic genes for that transformation are detected within a MAG, even when nonessential accessory genes are absent due to incomplete genome recovery. Dissimilatory nitrate reduction was the most prevalent nitrogen pathway, occurring in the cyanospheres of all but three cyanobacteria, *Trichormus* sp., *Plectolyngbya* sp., and *Aphanothece saxicola*. It was most abundant in *Kastovskya adunca* and *Desmonostoc vinosum* cyanospheres (Fig. 6). Assimilatory nitrate reduction was dominant in cyanospheres of *Tildeniella nuda* and *Nostoc desertorum*, overall appearing in 51 of 56 cyanospheres. The complete nitrification pathway was less common, detected in 28 cyanospheres, primarily in *Tildeniella nuda*, *Desmonostoc vinosum, Brasilonema angustatum,* and *Timaviella obliquedivisa*. Denitrification was present in 48 cyanospheres, most frequently in *Oscillatoria princeps* and *Desmonostoc vinosum* cyanospheres. Nitrate assimilation was observed in 50 cyanospheres, including cyanospheres of *Rexia erecta* and *Kastovskya adunca*. Complete nitrification (ammonia to nitrite to nitrate) was found in 28 cyanospheres. The conversion of nitrite and ammonia to nitrogen was found in 42 cyanospheres, particularly in *Tildeniella nuda* and *Desmonostoc vinosum*.

Interestingly, 15 cyanospheres contained nitrogen fixation genes, including the cyanospheres of *Iphinoe* sp., *Brasilonema octagenarum*, *Rexia erecta, Roholtiella mojavensis, Calothrix* sp.*, Goleter apudmare, Nostoc indistinguendum, Nostoc desertorum, Trichormus* sp.*, Scytonematopsis contorta, Brasilonema angustatum, Oscillatoria princeps, Timaviella radians, Pegethrix bostrychoides*, and “*Hassallia”* sp. The nitrogen fixation genes were associated with the following bacterial genera: *Sphingopyxis* (*Iphinoe* sp.), *Mycobacterium* (*Brasilonema octagenarum*), *Allorhizobium* (*Calothrix* sp.), *Sphingomonas* (*Goleter apudmare*), *Devosia* (*Nostoc desertorum*), *Agrobacterium* (*Nostoc indistinguendum*), *Nocardoides* (*Rexia erecta*), *Mesorhizobium* (*Trichormus* sp.), *Bradyrhizobium* (*Brasilonema angustatum*, *Oscillatoria princeps*), *Skermanella* (“*Hassallia*” sp.), *Tabrizicola* (*Pegthrix bostrychoides*), *Erythrobacter* (*Timaviella radians*) and unknown genera in *Scytonematopsis contorta* and *Roholtiella mojaviensis*.

This pattern contrasted with nitrogen-cycling pathways observed in the cyanobacterial genomes themselves. Assimilatory nitrate reduction, i.e., the reduction of nitrate to ammonium for incorporation into biomass as part of nitrate assimilation, was detected in 38 cyanobacteria, while complete nitrification was present in only five species (*Trichormus* sp., *Goleter apudmare*, *Scytonematopsis contorta*, *Brasilonema octagenarum*, and *Nostoc desertorum*). Denitrification was found in 27 cyanobacteria, and dissimilatory nitrate reduction, although the most abundant pathway in the cyanosphere, was observed in only 24 cyanobacteria. Nitrate assimilation (comprising nitrite uptake and its reduction via assimilatory nitrate reduction) was present in 32 cyanobacteria, whereas, similar to their associated cyanospheres, no cyanobacteria encoded pathways for the conversion of ammonia to nitrite. Nitrogen fixation pathways were detected in 25 cyanobacteria, including species that also hosted nitrogen-fixing heterotrophs in their cyanospheres (*Brasilonema octagenarum*, *Goleter apudmare*, *Nostoc desertorum*, *Scytonematopsis contorta*, *Brasilonema angustatum*, and “*Hassallia* sp.).

**Fig 7:**
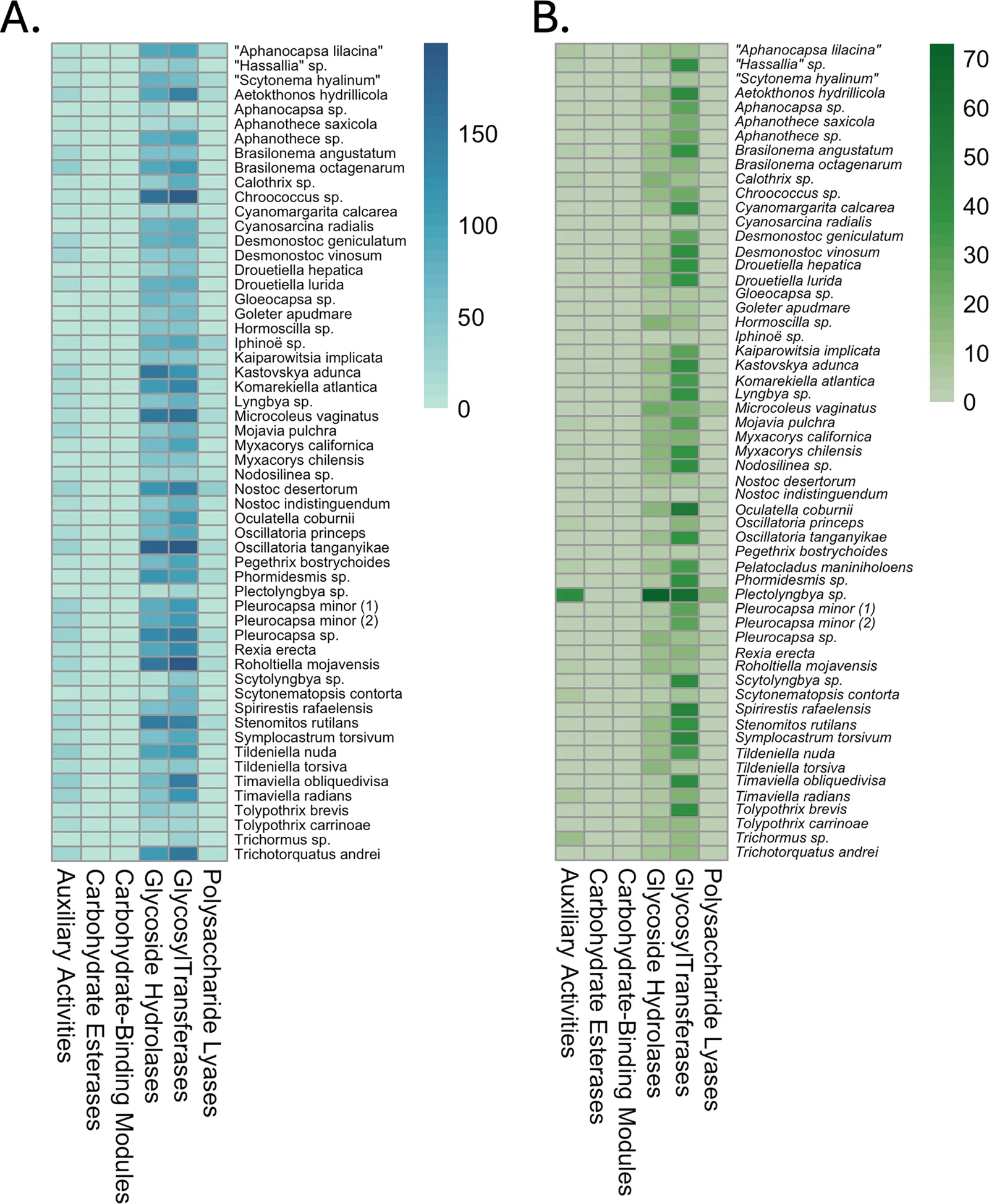
Heatmaps depicting the abundance of genes relating to CAZy pathways found in the cyanosphere (**A**) and the cyanobacteria (**B**). Pathway presence was defined according to DRAM’s marker-based criteria.

**Fig 8:**
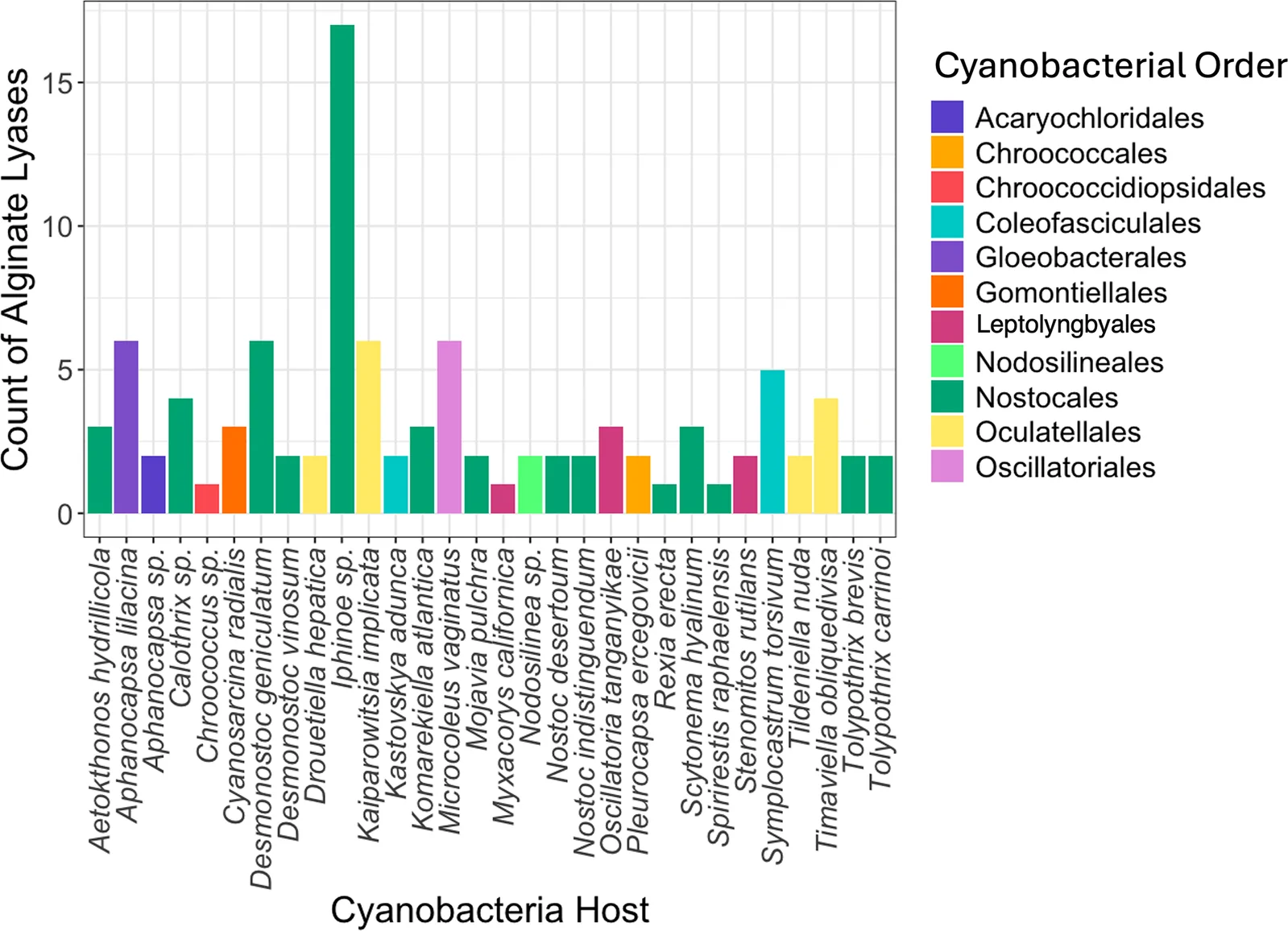
Total number of alginate lyases found in the cyanosphere of each cyanobacterial host. The colors represent the order of the cyanobacterial host.

Carbohydrate-Active enZYme (CAZy) pathways were prevalent in the cyanospheres, particularly glycosyl transferases and glycoside hydrolases (Fig. 7). The cyanospheres of “*Oscillatoria tanganyikae”* and *Chroococcus* sp. contained the highest number of genes related to glycoside hydrolases, while the cyanospheres of *Roholtiella mojaviensis* and “*Oscillatoria tanganyikae”* contained the most genes related to glycosyl transferases. The *Nostoc desertorum* cyanosphere contained the most polysaccharide lyase genes. Nine cyanospheres did not contain any genes relating to polysaccharide lyases including *Oculatella coburnii, Oscillatoria princeps, Goleter apudmare, Cyanomargarita calcarea, Aphanothece saxicola, Plectolyngba* sp.*, Scytonematopsis contorta, Trichormus* sp.*,* and *Tolypothrix carrinoi*. Interestingly, genes for carbohydrate-binding molecules and carbohydrate esterases were absent from all cyanospheres. Among the polysaccharide lyases identified, five alginate lyases were found: PL5, PL6, PL15, PL17. Thirty-two cyanospheres, mostly from Nostocales (*n* = 15), contained alginate lyases, while *Synechococcales* cyanospheres lacked these enzymes (Fig. 8). There was a significant difference in the number of alginate lyases between cyanospheres (ANOVA, *F* = 1.408, *P* = 0.0353). The *Iphinoe* sp. cyanosphere had the highest number of alginate lyases, followed by *Microcoleus vaginatus* (Fig. 8).

The CAZy genes present in the cyanosphere differed notably from those found in the cyanobacterial host. In the cyanobacteria, *Plectolyngbya* sp. had a high abundance of glycosyl transferase and glycoside hydrolase-related genes (Fig. 7). Polysaccharide lyases, however, were relatively scarce in cyanobacteria, with only 28 species containing these genes. Of those, only *Plectolyngbya* sp.*, Microcoleus vaginatus, Gloeocapsa* sp.*, Nostoc indistiguendum, Pleurocapsa* sp.*,* and *Rexia erecta* had more than two genes associated with polysaccharide lyases.

Additionally, we screened the MAGs for photosynthesis-related pathways using DRAM. Twenty-one MAGs encoded for complete oxygenic photosystems (PSI and PSII), including *Sphingomonas, Agrobacterium, Allorhizobium, Devosia, Flavobacterium, Mesorhizobium,* and *Sphingopyxis*. Thirty-nine MAGs coded for anoxygenic photosystem II, including *Brevundimonas, Roseomonas, Sphingopyxis,* and *Bosea*. All MAGs possessed photorespiration genes (Fig. S3).

## DISCUSSION

Using metagenomic sequencing, we identified non-cyanobacterial members of the cyanosphere from various habitats and cyanobacterial orders from cultured cyanobacteria. We characterized the cyanosphere membership for a broad range of terrestrial cyanobacteria, providing insight into interspecific interactions and the growth conditions necessary for cyanobacteria. The presence of core genera, such as *Brevundimonas, Devosia,* and *Sphingopyxis*, suggests that certain heterotrophs may routinely interact with the cyanobacterial hosts collected from a variety of habitats. Because cyanosphere composition was so strongly associated with environmental factors from which the cyanobacteria were collected, our findings further imply that successful culturing and ecological restoration efforts may depend on maintaining or recruiting microbial partners suited to the host’s native environmental conditions.

### Culturosphere vs cyanosphere

The terms *culturosphere* and *cyanosphere* are often used to distinguish between microbial communities associated with cultured organisms and those in natural environments. While these concepts are distinct, in practice, they may overlap in certain cases. Cyanobacteria, for example, are difficult to maintain in axenic conditions, largely due to their production and release of organic carbon, which readily supports the growth of heterotrophic bacteria (43–45). As a result, culturospheres can arise through two non-exclusive processes: (i) the retention and selective enrichment of a subset of the original, environmentally derived cyanosphere community under laboratory conditions and (ii) the introduction and colonization of additional, environmentally or laboratory-derived heterotrophs during cultivation. In addition, the composition of these communities is strongly shaped by culture conditions, including nutrient composition (e.g., nitrate availability), growth medium, and incubation regime, which impose selective pressures that can reduce diversity and lead to a culture-driven bottleneck effect (46–48). Consequently, cultures of cyanobacteria are typically co-cultured with allochthonous heterotrophs, and the line between culturosphere and cyanosphere can be blurred. In our study, the bacterial genera detected in the cyanobacterial cultures were consistent with those reported from the natural habitats (49–51), suggesting that the bacterial community present in our cultures mirrors at least a subset of the natural cyanosphere. Although we used MAGs from cultured cyanobacteria, the consistent differences in community composition among cultures originating from different cyanobacterial strains, as well as correlations with environmental parameters from the collection sites, suggest that the composition of the culturosphere retains a detectable signature of the original cyanosphere. This pattern implies that aspects of the host–microbe associations are preserved through isolation and maintenance in culture.

### Cyanosphere taxa are similar to known environmental genera

Based on 415 bacterial MAGs from 56 cyanobacterial hosts, we identified patterns between the cyanospheres. Members of the cyanosphere recovered include many genera found in terrestrial ecosystems where the cyanobacteria were found, including soils, biological soil crusts, microbial mats, and environmental biofilms, such as *Brevundimonas* and *Sphingomonas*, which are frequently recovered from biological soil crusts and oligotrophic soils (49, 50, 52–55). Other genera detected including *Devosia, Variovorax, Mesorhizobium, Bradyhizobium,* and *Pararhizobium* are common soil and rhizosphere taxa important for nutrient cycling and plant-associated interactions (51, 56–63), underscoring the potential for nutrient exchanges in the cyanosphere. Members of *Flavobacterium* and related Bacteroidota are prominent degraders of complex polysaccharides (64–66), were also present, and could contribute to exopolysaccharide turnover. Other soil and biofilm associated heterotrophs recovered here include *Paenibacillus, Nocardoides, Pseudomonas, Sphingopyxis, Microvirga,* and *Methylobacterium*, each of which has been reported from environmental biofilms, soils, or biocrusts (67–79).

### Trends within cyanospheres

The most common genera, found in over 30% of the cyanospheres, were *Brevundimonas*, *Devosia*, and *Sphingopyxis*. These genera might indicate a “core microbiome,” as prior studies have used abundance in 30% of samples as an arbitrary cutoff for calculating core communities (80, 81). The genus *Brevundimonas,* found in 37.5% of the cyanospheres, is broadly distributed across diverse environments, including plant-root zones, activated sludge, alkaline soils, and thermal water (82–88). This broad ecological range suggests that *Brevundimonas* lineages are common in nature, consistent with their presence in cyanosphere communities derived from environmentally collected cyanobacteria.

*Devosia* is frequently detected in soils and aquatic sediments (56, 89–92) and was also relatively common in our data set, occurring in 36% of cyanospheres and accounting for 5.6% of all recovered MAGs. Certain *Devosia* species are capable of forming nitrogen-fixing root-nodule symbioses with plants (51). In this study, we found nitrogen-fixing genes in *Devosia* within the *Nostoc desertorum* cyanosphere despite *N. desertorum* being capable of nitrogen fixation itself. *Devosia* have also been reported from phototroph-associated niches, including the phycosphere of marine red algae (93). Previous genomic work highlights metabolic and genomic plasticity across the genus (94), suggesting that *Devosia* lineages have broad ecological potential.

*Sphingopyxis* occurred in 30% of the cyanobacterial hosts and represented 4.4% of all cyanosphere MAGs. Across environmental studies, this genus is reported from a wide range of habitats, including aquatic sediments (95), contaminated soils (96, 97), and activated sludge (98). Several species within the genus can degrade environmental contaminants and produce secondary metabolites (99), traits that may contribute to their persistence in stressful conditions.

The richness and diversity of the microbial community did not vary significantly between the cyanospheres but cyanosphere composition was influenced by the cyanobacterial host. A previous study with the rhizosphere showed that bacterial richness and diversity were greater in the bulk soil compared to the rhizosphere and that the richness and diversity of the rhizosphere rarely varied between rhizospheres (100). The authors state that the rhizosphere microbiota have similar traits regardless of the plant, thus resulting in similar richness and diversity measurements regardless of the phylogenetic placement of the bacteria. This similarity in richness and alpha-diversity across cyanospheres may reflect the recruitment of bacteria fulfilling comparable functional roles across hosts, although stochastic assembly and founder effects followed by functional filtering during culture establishment remain equally plausible explanations.

### Habitat influences the cyanosphere microbiome

The community composition of the cyanosphere was influenced more by the habitat of the host origin than the phylogeny of the cyanobacteria host. This suggests that the microbes forming the cyanosphere are not determined by relationships between the cyanosphere and the phylogenetic affiliation of the host cyanobacteria at the broader taxonomic level.

The effect of habitat could be due to recruitment of the cyanosphere microbiome from the surrounding microbial community. This commonly occurs within the rhizosphere, where the rhizosphere microbiome is a specific subset of the bulk soil microbiome (101). In the rhizosphere, root traits can explain the community composition (102). Thus, in the cyanosphere, traits of the cyanobacteria such as the chemical composition and physical characteristics of the exopolysaccharides could be the factors that determine which bacteria colonize the cyanosphere.

One of the limits of this data set is the lack of even sampling across locations. This should be rectified in future studies. As such, there was a bias between the habitat of host origin and the sampling location. For example, 21 of the 29 soil cyanospheres were collected from North America (primarily Mojave and Sonoran Deserts, Southern California). Thus, it is difficult to parse out if the difference in the community composition between habitats is due to the effect of the particular habitat or due to the effect of sampling location, in this case, with a bias toward microbial communities that are present in California. The problem is not persistent within the other habitat types, as the subaerial samples (22) have 3 from Europe, 10 from North America, and 9 from the Pacific Islands, and the freshwater samples are from Europe, North America, and the Pacific Islands. Furthermore, there was uneven sampling across habitats with 29 samples from the soil, 22 subaerial, 3 freshwater, and 2 hydroterrestrial (both from Hawaii). This should not discount the results presented in our exploratory study thus far, but rather should be used as a stepping stone for subsequent research. Given the differences in the microbial community composition between habitats (or locations), there is a need for further research to determine the specific factors that influence the cyanosphere microbial community composition.

### Cyanospheres and cyanobacteria differ in predicted nitrogen and carbon exchange pathways

Nitrogen metabolism is a fundamental component of cyanobacterial ecology and influences interactions within the cyanosphere (13). Cyanobacteria release organic carbon that stimulates heterotrophic activity, promoting processes such as nitrogen remineralization and ammonification that can support growth (13, 103). These pathways provide ecological context for potential nutrient exchange between cyanobacteria and their associated heterotrophs and enable comparison with other studies in which nitrogen metabolism is considered a core function of cyanobacterial consortia.

The detection of nitrogen fixation genes in a subset of cyanosphere members highlights the potential for heterotrophic bacteria to contribute to nitrogen availability within these microbial consortia. In our data set, 15 MAGs had nitrogen fixation genes in their recovered genomes, including *Sphingopyxis*, *Mycobacterium*, *Allorhizobium*, *Sphingomonas*, *Devosia*, *Agrobacterium*, *Nocardoides*, *Mesorhizobium*, *Bradyrhizobium*, *Skermanella*, *Tabrizicola*, and *Erythrobacter*. Diazotrophy was present in some cyanobacterial hosts as well. The presence of nitrogen-fixing capabilities in both the hosts and the cyanosphere suggests that nitrogen acquisition in cyanobacteria is not solely dependent on the host but may also be supported by symbiotic or opportunistic interactions with associated diazotrophs. Previous cyanosphere studies have emphasized the role of nitrogen-fixing cyanosphere members for non-diazotrophic hosts (13, 14, 25, 26). For example, heterotrophic diazotrophs may supplement nitrogen fixation when cyanobacterial activity is limited by environmental stressors (14), such as desiccation, nutrient limitation, or fluctuating light conditions. In nutrient-poor terrestrial soils, this redundancy could improve resilience (104). Conversely, nitrogen transfer within the cyanosphere may be spatially or physiologically constrained, meaning that fixed nitrogen is not always readily available to all community members (13, 26). Under such conditions, associated microbes may need to rely on their own nitrogen fixation capacity to sustain growth.

However, the communities may have also been influenced by the culture medium. The composition of Z8 medium, in which the cultures were maintained (2), has a high concentration of nitrate and a lower concentration of ammonium, which may exert selective pressure on the microbial communities in culture. However, it is also important to consider the potential role of the habitat from which the cyanobacteria were collected. In their natural environments, cyanobacteria are exposed to different nitrogen sources and may already harbor microbial communities that have adapted to utilize these compounds (105). Therefore, while culture conditions play a role in shaping the microbiome, the microbial traits observed may also reflect ecological characteristics shaped by the natural environment of the cyanobacteria (22, 106).

Similarly, the detection of photosynthesis-related pathways among cyanosphere MAGs indicates that phototrophic metabolism is not limited to the cyanobacterial hosts but is also represented among associated community members, including abundant cyanosphere members such as *Sphingomonas, Devosia,* and *Sphingopyxis*. While complete oxygenic photosystems were restricted to a subset of MAGs, the widespread presence of anoxygenic photosystem II suggests that alternative light-driven energy acquisition strategies may be common within the cyanosphere. Such metabolic versatility could allow cyanosphere bacteria to exploit light in microenvironments shaped by cyanobacterial activity, particularly in terrestrial systems where light availability and redox conditions fluctuate. However, the presence of genes coding for PSI and PSII does not necessarily prove the organism is capable of autotrophy.

There were also complementary patterns between the cyanosphere and cyanobacterial hosts within the CAZy framework. The widespread presence of glycosyl transferases and glycoside hydrolases, as well as the abundance of polysaccharide lyases (PLs), suggests that cyanosphere bacteria are able to modify and degrade complex carbohydrates produced by the cyanobacterial hosts. This is notable since the cyanobacteria themselves have fewer polysaccharide-degrading enzymes, supporting the idea that cyanobacterial extracellular polymeric substances (EPS) represent a primary carbon source for cyanosphere metabolism (107, 108). Additionally, EPS plays a central role in terrestrial systems, where it contributes to carbon storage, soil aggregation, and moisture retention (108).

Polysaccharide lyases (PLs), which can degrade EPS as a carbon source, were found in 86% of the cyanospheres (Fig. 7). This breadth contrasts with global soil and plankton microbiomes, where PLs occur at lower prevalence (109, 110). This suggests that cyanosphere bacteria may be adapted for metabolizing cyanobacterial exopolysaccharides, consistent with the known role of EPS as a major carbon source in terrestrial cyanobacteria (111).

The PLs present in the cyanosphere were mostly composed of alginate lyases (Fig. 8). Alginate is a common component of cyanobacterial and algal EPS, and the presence of multiple alginate lyase families (including PL5, PL6, PL15, and PL17) indicates that cyanosphere bacteria possess strategies for alginate degradation. The association of alginate lyases with Nostocales cyanospheres, and their absence from Synechococcales cyanospheres, suggests that host identity and EPS composition may influence the functional structure of the cyanosphere.

Key cyanosphere genera, *Sphingomonas* and *Brevundimonas,* contain polysaccharide lyases. *Sphingomonas* strains can synthesize gellan lyase (112) and alginate lyases (113). *Sphingomonas* with polysaccharide lyase genes was found in 7 of the cyanospheres and was present in all terrestrial environments (1 subaerial, 1 hydroterrestrial, 5 soil). Perhaps its ability to degrade EPS contributes to its prevalence in all three terrestrial habitats. *Brevundimonas*, part of the “core” cyanosphere, also contained several polysaccharide lyases within 28% of the cyanospheres, with the greatest abundance in *Kastovskya adunca, Mojavia pulchra,* and *Nodosilinea* sp. A particular group of polysaccharide lyases—alginate lyases—can degrade alginate, a polysaccharide found in algae and cyanobacteria (114, 115). The cyanosphere contained six types of alginate lyases (PL6 alginate lyase, PL5 alginate lyase, M-specific alginate lyase, PL15 oligo-alginate lyase, PL17 alginate lyase, PL14 alginate lyase). In contrast, polysaccharide lyases were relatively rare in cyanobacterial genomes and, when present, were typically represented by only one or two genes. This disparity between host and cyanosphere CAZy repertoires highlights a potential division of labor, in which cyanobacteria invest in EPS production while associated heterotrophs specialize in its modification and turnover.

### Implications

The application of terrestrial cyanobacteria in ecosystem restoration is an emerging and rapidly expanding field (116–118). These microorganisms play a role in dryland ecosystem restoration due to their remarkable ability to withstand environmental stressors and the various ecological functions they support (119). Cyanobacteria contribute to critical processes such as stabilizing soils, enhancing soil fertility, promoting nutrient cycling, increasing water retention and infiltration, and sequestering carbon (117, 118, 120, 121). These functions are essential for restoring degraded environments, particularly in arid and semi-arid regions where soil erosion, nutrient depletion, and poor water retention are prevalent challenges.

The success of restoration efforts involving cyanobacteria can be closely tied to the cyanosphere (25). The composition of the cyanosphere may play a role in determining the effectiveness of cyanobacteria in restoration activities. Therefore, it is essential (i) to investigate the relationships between cyanosphere microbes and their host more deeply and (ii) to consider the unique characteristics and requirements of the cyanosphere when selecting and applying cyanobacteria in ecosystem restoration projects, to optimize their performance and ensure long-term ecological benefits.

## Data Availability

The sequence reads for the project are deposited under BioProject PRJNA1231202 (Table S1).

## SUPPLEMENTAL MATERIAL

Fig. S1 aem.01043-26-s0001.png Plot of the genera identified using unassembled reads and Kaiju, providing an assembly-independent overview of the community composition.

Fig. S2 aem.01043-26-s0002.png PcOA plot based on the unassembled reads identified with Kaiju.

Fig. S3 aem.01043-26-s0003.png Heatmap of the abundance of photosynthesis genes based on the analysis with DRAM within each cyanosphere.

Supplemental legends aem.01043-26-s0004.docx Captions for supplemental material.

Table S1 aem.01043-26-s0005.xlsx Metadata for the cyanobacteria used in this study.

Table S2 aem.01043-26-s0006.csv Summary statistics from each MAG, including completeness, contamination, number of contigs, genome size, and taxonomic information.

Table S3 aem.01043-26-s0007.csv Abundance of photosynthesis genes identified in each genus.

ASM does not own the copyrights to Supplemental Material that may be linked to, or accessed through, an article. The authors have granted ASM a non-exclusive, world-wide license to publish the Supplemental Material files. Please contact the corresponding author directly for reuse.
