# Supplementary material for: Unraveling the diversity and functional potential of cyanosphere microbiomes assembled from terrestrial cyanobacteria
Source: Appl Environ Microbiol. 2026 Jul 20;92(8):e01043-26. doi: 10.1128/aem.01043-26 (PMC13488342; doi:10.1128/aem.01043-26)
Supplement: Supplementary file 1 [file aem.01043-26-s0004.docx]

**Supplementary**

**Table S1:** Metadata for the cyanobacteria used in this study. The “Map ID” column corresponds with the locations of the cyanobacteria depicted in Figure 1. The annual mean temperature and annual precipitation were derived from BioClim data when accurate latitude and longitude coordinates were provided for the cyanobacterial hosts. The * denote cyanobacteria that were not previously published in Ward et al. 2021.

**Table S2:** Summary statistics from each MAG including completeness, contamination, number of contigs, genome size, and taxonomic information.

**Table S3:** Abundance of photosynthesis genes identified in each genera.

**Figure S1:** Kaiju Plot: A plot of the genera identified using unassembled reads and Kaiju, providing an assembly-independent overview of the community composition.

**Figure S2:** Kaiju PcOA: A PcOA plot based on the unassembled reads identified with Kaiju. Each point represents the cyanosphere of one cyanobacterial host. The points are color-coded based on the habitat where the cyanobacterial host was collected from.

**Figure S3:** Photosynthesis: A heatmap of the abundance of photosynthesis genes based on the analysis with DRAM within each cyanosphere. Red indicates high abundance while blue indicates low to no abundance.
